# Off-the-Shelf Gd(NO_3_)_3_ as an Efficient High-Spin Metal Ion Polarizing Agent for Magic Angle Spinning Dynamic Nuclear Polarization

**DOI:** 10.1021/acs.jpcb.2c04184

**Published:** 2022-08-16

**Authors:** Stuart J. Elliott, Benjamin B. Duff, Ashlea R. Taylor-Hughes, Daniel J. Cheney, John P. Corley, Subhradip Paul, Adam Brookfield, Shane Pawsey, David Gajan, Helen C. Aspinall, Anne Lesage, Frédéric Blanc

**Affiliations:** † Department of Chemistry, University of Liverpool, Liverpool L69 7ZD, United Kingdom; § Stephenson Institute for Renewable Energy, University of Liverpool, Liverpool L69 7ZD, United Kingdom; ∥ DNP MAS NMR Facility, Sir Peter Mansfield Imaging Centre, University of Nottingham, Nottingham NG7 3RD, United Kingdom; △ Department of Chemistry and Photon Science Institute, University of Manchester, Oxford Road, Manchester M13 9PL, United Kingdom; ▽ Bruker BioSpin Corporation, Billerica, Massachusetts 01821, United States; □ Université de Lyon, Centre de Résonance Magnétique Nucléaire à Très Hauts Champs (UMR 5082, CNRS/ENS Lyon/UCBL), 69100 Villeurbanne, France

## Abstract

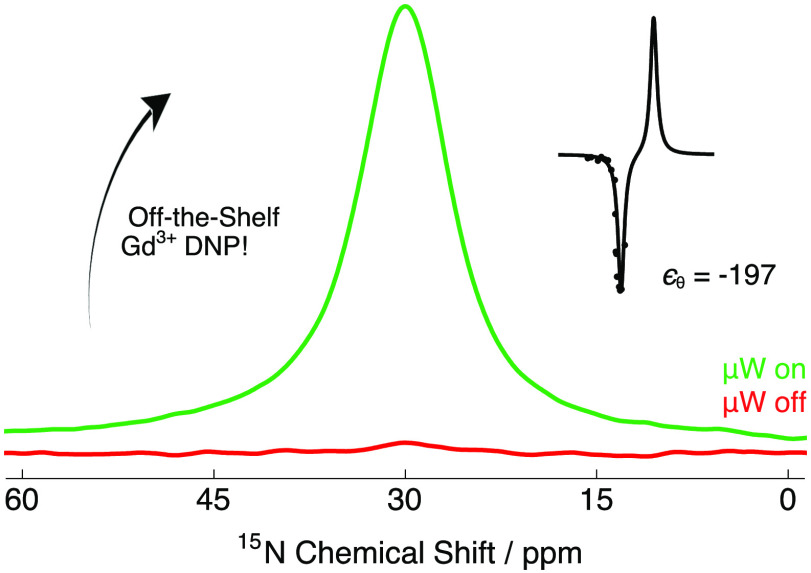

Magic angle spinning nuclear magnetic resonance spectroscopy experiments are widely employed in the characterization of solid media. The approach is incredibly versatile but deleteriously suffers from low sensitivity, which may be alleviated by adopting dynamic nuclear polarization methods, resulting in large signal enhancements. Paramagnetic metal ions such as Gd^3+^ have recently shown promising results as polarizing agents for ^1^H, ^13^C, and ^15^N nuclear spins. We demonstrate that the widely available and inexpensive chemical agent Gd(NO_3_)_3_ achieves significant signal enhancements for the ^13^C and ^15^N nuclear sites of [2-^13^C,^15^N]glycine at 9.4 T and ∼105 K. Analysis of the signal enhancement profiles at two magnetic fields, in conjunction with electron paramagnetic resonance data, reveals the solid effect to be the dominant signal enhancement mechanism. The signal amplification obtained paves the way for efficient dynamic nuclear polarization without the need for challenging synthesis of Gd^3+^ polarizing agents.

## Introduction

Magic angle spinning (MAS) nuclear magnetic resonance (NMR) spectroscopy is a very powerful approach to probe the atomic-scale structure, dynamics, and function of materials and proteins in the solid state.^1−3^ However, such experiments are often limited by the intrinsically weak NMR signal response of nuclear spin ensembles at room temperature even at the highest field of currently available superconducting NMR magnets. Dynamic nuclear polarization (DNP) provides a solution to overcome this limitation^4−6^ by transferring the high polarization of electron spins to weakly polarized nuclear spins followed by detection of enhanced NMR signals. This method has been successfully exploited in MAS NMR experiments in the context of structural biology,^7^ materials science,^8,9^ catalysis,^10−12^ pharmaceutical science,^13,14^ and other areas.^15−17^

DNP achieves sizable NMR signal enhancements ϵ by: (i) utilizing formulated samples that are composed of aqueous/organic and glass-forming solvents doped with optimal concentrations of exogenous paramagnetic polarizing agents (PAs) and the spin system of interest; (ii) freezing the DNP-compatible sample at low temperature (ca. 105 K); and (iii) irradiating the sample with a microwave field that is typically resonant or marginally nonresonant with respect to the electron spin transition frequency. Under these conditions, the last step transfers the high electron spin polarization to the weakly polarized nuclear spins of choice.^5,18^

Tremendous advances have been achieved in DNP at fast-MAS rates,^19^ in high magnetic fields,^20−22^ and in the design of efficient nitroxide-based biradicals as PAs for MAS DNP such as TOTAPOL,^23^ bTbk,^24^ AMUPol,^25^ TEKPol,^26^ AsymPol,^27^ TinyPols,^28^ and others.^29^ For example, TOTAPOL biradicals have been bound to functional amyloid fibril surfaces, which allowed the collection of enhanced NMR spectra using significantly reduced PA concentrations;^30^ an organometallic complex supported on a hydrophobic surface was characterized using enhanced MAS NMR signals from DNP employing water-insoluble bTbk in a combination of nonaqueous solvents;^31^ bTbk was later placed into DNP juice (H_2_O/D_2_O/glycerol-*d*_8_, 1/3/6 v/v/v) with the use of a surfactant;^32^ TEKPOL PAs dissolved in a glassy phase of *ortho*-terphenyl led to ^1^H enhancements surpassing 80 at 240 K, enabling monitoring of molecular dynamic transitions in pharmaceutically relevant solids.^33^ Similar enhancements were found using the hybrid trityl- or BDPA-nitroxide biradicals TEMTriPol^34^ or HyTEK2,^35^ respectively. Nevertheless, several potential limitations for these PAs exist and include sample formulation, chemical incompatibilities (e.g., acidic substrates,^36^ reducing environments^37^), critical availability and accessibility due to their structural complexities, and resulting challenging chemical synthesis (very few are available commercially, and most can only be prepared from lengthy, nontrivial, and poor-yielding synthetic routes).

More recently, inspired by paramagnetic transition metal and lanthanide complexes used for *in vivo* magnetic resonance imaging (MRI) applications,^38,39^ high-spin metal ions have shown potential^40−44^ as alternatives to tailored nitroxide-based biradical PAs for specific applications, ^1^H DNP, or the direct DNP of lower gamma nuclear spins. Gd^3+^ is of particular interest, as it offers large ^1^H NMR signal enhancements ϵ of ∼19 for [Gd(dota)(H_2_O)]^−^ and ∼37 for [Gd(tpcatcn)] complexes at 9.4 T.^43^ High-spin metal ions also allow for endogenous hyperpolarization where intrinsic ions are contained in the samples as dopants (in inorganic solids) such as Mn^2+^-doped Li_4_Ti_5_O_12_^45^ or (in oxide glasses) such as Gd^3+^-doped lithium silicate, lithium borate, and zinc phosphate^46^ for bulk signal enhancements, in Gd^3+^-doped CeO_2_ nanocomposite thin films on SrTiO_3_ for detailed study of material interfaces,^47^ and in spin-labels as chelator tags.^41^

An exciting opportunity for MAS DNP consists of utilizing an “off-the-shelf” paramagnetic metal ion species as a PA that is easily affordable and does not require any chemical synthesis. Here, we show that a PA obtained by using commercially available Gd(NO_3_)_3_ as the Gd^3+^ ion source can efficiently hyperpolarize heteronuclear spins, and we report direct NMR signal enhancements ϵ of −16 (^13^C) and *–*57 (^15^N) and direct overall NMR signal enhancements^48^ ϵ_θ_ of −35 (^13^C) and −197 (^15^N) at 9.4 T and ∼105 K. While Gd complexes stabilized by organic ligands such as [Gd(tpcatcn)]^43^ yield higher enhancements ϵ, this work demonstrates that a simpler, more stable, and readily available Gd^3+^ source from Gd(NO_3_)_3_ is also very efficient, most notably for ^13^C and ^15^N nuclear spins. It should also be noted, however, that GdCl_3_, another off-the-shelf PA, has previously been used in DNP experiments in comparison with other Gd complexes.^41^ Scrutiny of the experimental signal enhancement profiles at two magnetic fields, supported by data from electron paramagnetic resonance (EPR) measurements, unveils the solid effect (SE) as the dominant polarization transfer mechanism.

## Methods

### Sample Preparation

A solution of 1.5 M [2-^13^C,^15^N]glycine (Sigma-Aldrich, 99% ^13^C, 98% ^15^N) doped with 20 mM Gd(NO_3_)_3_·6H_2_O (Sigma-Aldrich, 99.99%) was prepared in the glass-forming mixture H_2_O/D_2_O/glycerol-*d*_8_ (1/3/6 v/v/v) for DNP experiments (note that the hexahydrate form of Gd(NO_3_)_3_ does not alter the ^1^H concentration significantly). An enriched sample was used to facilitate measurements of ^13^C and ^15^N NMR signal enhancements. Samples were sonicated at 65 °C for 15 min to ensure complete dissolution. A 15–25 μL amount of solution was packed into a 3.2 mm sapphire rotor and closed with a silicone plug and a Vespel drive cap. A solution of 20 mM Gd(NO_3_)_3_·6H_2_O was prepared in the glass-forming mixture H_2_O/glycerol (2/3 v/v) for EPR experiments. EPR tubes with outer diameters of 4 mm (X-band) and 3 mm (Q-band) were filled with the solution to a 1 cm height to ensure complete coverage of the active region of the resonator. Gd(NO_3_)_3_·6H_2_O and GdCl_3_·6H_2_O (Alfa Aesar, 99.99%) were dissolved in H_2_O/glycerol (2/3 v/v) (with a small amount of HCO_2_H to ensure complete dissolution) for HRMS experiments.

### DNP MAS NMR

Experiments were performed on a commercial Bruker Biospin DNP system^49^ at a static magnetic field *B*_0_ = 9.4 T on a 400 MHz AVANCE III HD spectrometer with a gyrotron microwave source operating at a frequency ω_0S_/2π = 263 GHz and at *B*_0_ = 14.1 T on a 600 MHz AVANCE III spectrometer at ω_0S_/2π = 395 GHz. (The ^1^H Zeeman field profile given in Figure S1 in the Supporting Information was obtained on a 400 MHz AVANCE III HD spectrometer but at a slightly lower *B*_0_ field, which shifts the upper limits of *B*_0_, and so the positive maximum is also observable.) Experiments were performed on 3.2 mm triple resonance HXY low-temperature MAS probes tuned to X = ^13^C and Y = ^15^N and at a MAS rate ω_r_/2π = 10 kHz. Field sweep experiments were obtained by varying *B*_0_ using the sweep coil of the Ascend DNP NMR magnet while keeping ω_0S_/2π constant. ^1^H, ^13^C, and ^15^N NMR spectra were obtained with a rotor-synchronized Hahn echo radiofrequency (*rf*) pulse sequence (^1^H data shown in Figure S1) and NMR signal buildup times of constants of 10, 30, and 40 s, respectively. *rf*-pulse amplitudes at 9.4 T: ω_H_/2π = 100 kHz, ω_C_/2π = 46 kHz, and ω_N_/2π = 40 kHz. *rf*-pulse amplitudes at 14.1 T: ω_H_/2π = 66 kHz, ω_C_/2π = 60 kHz, and ω_N_/2π = 38 kHz. ^1^H → ^13^C CP NMR spectra were collected by matching to the Hartmann–Hahn condition ω_H_ = ω_C_ ± *n*ω_r_, where *n* is an integer with a 70% → 100% linear amplitude ramp for ω_H_, a CP contact duration of 0.5 ms (9.4 T) or 2 ms (14.1 T), and a repetition delay set to 1.3 × *T*_B,ON_(^1^H) = 4.3 s as measured below were used. ^1^H → ^13^C CP *rf*-pulse amplitudes at 9.4 T: ω_H_/2π = 80 kHz and ω_C_/2π = 39 kHz. ^1^H → ^13^C CP *rf*-pulse amplitudes at 14.1 T: ω_H_/2π = 50 kHz and ω_C_/2π = 60 kHz. SPINAL-64 decoupling^50^ was applied during ^13^C and ^15^N NMR signal acquisition with a ^1^H *rf*-pulse amplitude of 100 kHz (9.4 T) or 66 kHz (14.1 T). A train of presaturation *rf*-pulses was employed on all relevant spectrometer *rf*-channels before commencing experiments. DNP buildup time constants *T*_B,ON_ (listed in Tables 1 and S1) were measured by saturating all starting magnetization, detecting the magnetization recovery at known time intervals, and fitting the resulting data with a stretched exponential function of the type *A*(1 – exp{−(*t*/*T*_B,ON_^*^)^α^}), where *A* is a fitting constant, *T*_B,ON_ = *T*_B,ON_^*^Γ(1/α)/α, *T*_B,ON_^*^ is the DNP buildup time constant extracted from the above-described fitting procedure, α is the breadth of the distribution of DNP buildup time constants, and Γ(1/α) is the gamma function (all fitted parameters are given in Table S1. Nuclear spin–lattice relaxation time constants *T*_1_ (Table S2) were measured on a sample without 20 mM dissolved Gd(NO_3_)_3_·6H_2_O by ^1^H → ^13^C/^15^N CP saturation recovery experiments. ^1^H relaxation data were fit with an exponential function of the type *B*(1 – exp{−*t*/*T*_1_}), where *B* is a fitting constant. ^13^C/^15^N relaxation data were fit with a biexponential function of the type *C* exp{−*t*/*T*_1_} + *D* exp{−*t*/*T*_i,r_}, where *C* and *D* are fitting constants and *T*_i,r_ accounts for an initial, rapid decay of nuclear magnetization. All data are reported at the optimum of the DNP enhancements as a function of the μW power curve. The NMR signal enhancement ϵ is defined as *I*_on_/*I*_off_, where *I*_on_ and *I*_off_ are the measured NMR spectral integrations in cases of μW on and off, respectively. The overall NMR signal enhancement ϵ_θ_ is defined in eq 1 below and depends on ϵ, *T*_B,ON_, *T*_1_, and the bleaching factor θ. θ is defined as *I*_with_/*I*_without_, where *I*_with_ and *I*_without_ are the measured ^1^H NMR spectral integrations in cases of samples with and without 20 mM Gd(NO_3_)_3_·6H_2_O, respectively. ^1^H, ^13^C, and ^15^N NMR spectra were externally referenced to the silicone plug at 0 ppm (both ^1^H and ^13^C) and the free amino acid in arginine at 37 ppm, respectively. The sample temperature was ∼105 K as determined by measuring *T*_1_(^79^Br) from saturation recovery experiments.^51^

**Table 1:** ϵ, ϵ_θ_, *T*_B,ON_, and  for ^1^H, ^13^C, and ^15^N Nuclear Spins at 9.4 and 14.1 T^a^

	ϵ			ϵ_θ_^b^			*T*_B,ON_/s					
*B*_0_/T	^1^H	^13^C	^15^N	^1^H	^13^C	^15^N	^1^H	^13^C	^15^N	^1^H	^13^C	^15^N
9.4	–2.6	–16	–57	–8.5	–35	–197	3.6 ± 0.1	165 ± 25	240 ± 11	–1.37 ± 0.02	–1.25 ± 0.08	–3.68 ± 0.08
14.1	–0.5	–11	–23	–1.5	–20	–68	14 ± 2	222 ± 7	304 ± 31	–0.13 ± 0.02	–0.74 ± 0.02	–1.3 ± 0.1

a 1.5 M [2-^13^C,^15^N]glycine doped with 20 mM Gd(NO_3_)_3_·6H_2_O in the glass-forming mixture H_2_O/D_2_O/glycerol-*d*_8_ (1/3/6 v/v/v ratio) at ∼105 K was used.

b Data at 14.1 T uses values of *T*_1_ measured at 9.4 T.

### EPR

EPR spectra at X-band (9.5 GHz) and Q-band (34 GHz) and 100 K were collected on Bruker E580 Elexsys pulsed spectrometers equipped with a Bruker 4118X-MD5 Flexline resonator (X-band) or a Bruker QT-II resonator (Q-band). Cryogenic temperatures were achieved with closed cycle cryofree cryostats from Bruker Biospin and Cryogenic Ltd. Echo-detected field-swept EPR spectra were recorded using a standard Hahn echo sequence of π/2−τ–π where π = 32 ns, τ = 180 ns (X-band) or 300 ns (Q-band). Electron transverse relaxation time constants *T*_2e_ (Table S3) were obtained with the same Hahn echo sequence with τ increasing in 2 ns increments. *T*_2e_ relaxation time constant data were fit with an exponential function of the type *E* exp{−(*t*/*T*_2e_)}, where *E* is a fitting constant. Electron longitudinal relaxation time constants *T*_1e_ (Table S1) were obtained using a three-pulse inversion recovery echo sequence π–*T*–π/2−τ–π where *T* = 100 ns. *T*_1e_ relaxation time constant data were fit with a stretched exponential function of the type *F*(1 – exp{−(*t*/*T*_1e_)^β^}), where *F* is a fitting constant, *T*_1e_ = *T*_1e_^*^Γ(1/β)/β, *T*_1e_^*^ is the electron longitudinal relaxation time constant extracted from the above-described fitting procedure, β is the breadth of the distribution of longitudinal relaxation time constants, and Γ(1/β) is the gamma function. A two-step phase cycle was employed to minimize effects from unwanted echoes. Simulations of echo-detected EPR spectra were produced using EasySpin^52^ and MatLab scripts from the ETH zero-field splitting (ZFS) library available at https://www.epr.ethz.ch/software.

### HRMS

HRMS data were recorded on an Agilent 6540 quadrupole-time-of-flight mass spectrometer using electrospray ionization in positive mode. For Gd(NO_3_)_3_·6H_2_O, *m*/*z*: calculated for [GdO]^+^ 173.9190, found 173.9185; calculated for [C_6_H_14_GdO_6_]^+^ (i.e., [Gd^3+^ + 2 × glycerol – 2 × H^+^]^+^) 340.0031, found 340.0026; calculated for [C_6_H_15_GdNO_9_]^+^ (i.e., [Gd^3+^ + 2 × glycerol + NO_3_^–^ – H^+^]^+^) 402.9988, found 402.9981. For GdCl_3_·6H_2_O, *m*/*z*: calculated for [GdO]^+^ 173.9190, found 173.9188; calculated for [C_6_H_14_GdO_6_]^+^ (i.e., [Gd^3+^ + 2 × glycerol – 2 × H^+^]^+^) 340.0031, found 340.0034.

## Results

**Figure 1:**
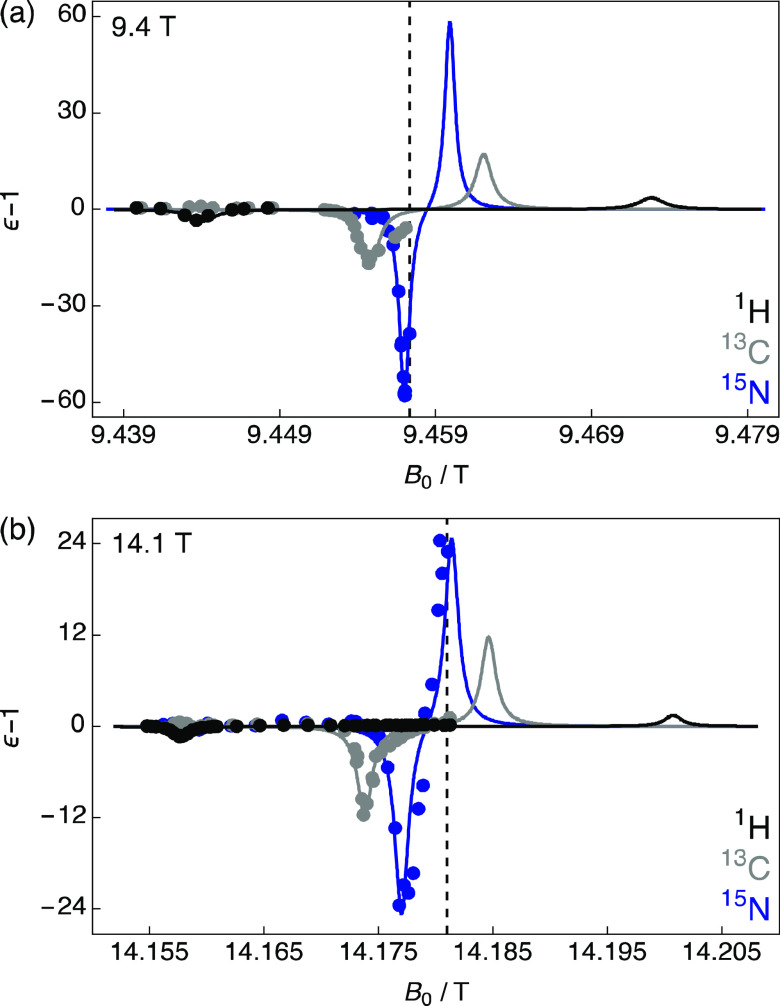
Normalized experimental ^1^H via ^13^C CP (black), direct ^13^C (gray), and direct ^15^N (blue) DNP MAS NMR Zeeman field profiles of 1.5 M [2-^13^C,^15^N]glycine doped with 20 mM Gd(NO_3_)_3_·6H_2_O dissolved in H_2_O/D_2_O/glycerol-*d*_8_ (1/3/6 v/v/v) as a function of the static magnetic field (*B*_0_) acquired at (a) 9.4 T and (b) 14.1 T and ∼105 K. The vertical axes are given as normalized enhancements (ϵ – 1). Solid lines are theoretical curves (see eq 3 and the main text for more details). Vertical dashed lines indicate upper limits of *B*_0_ due to the maximum current permitted in the sweep coil on each spectrometer. Figure S2 shows magnified views of the ^1^H enhancements.

Figure 1 shows the normalized experimental ^1^H via ^13^C CP (black), direct ^13^C (gray), and direct ^15^N (blue) DNP MAS NMR Zeeman field profiles as a function of the static magnetic field (*B*_0_) at 9.4 T (Figure 1a) and 14.1 T (Figure 1b) under 10 kHz MAS at ∼105 K for 20 mM Gd(NO_3_)_3_·6H_2_O (typical PA concentration) in H_2_O/D_2_O/glycerol-*d*_8_ in a 1/3/6 v/v/v ratio (standard aqueous glass forming matrix, so-called DNP juice) as measured on [2-^13^C,^15^N]glycine (further details are given in the Methods section). At these magnetic fields, ^1^H nuclear spins display no considerable NMR signal enhancement under microwave irradiation with ϵ = −2.6 and −0.5 obtained at 9.4 and 14.1 T (Figures 1, S1 and S2), respectively. Slight NMR signal enhancements for ^13^C nuclear spins are observed at the negative lobe of the ^1^H NMR signal enhancement profile, but with opposite phase that corresponds to a magnetization transfer due to heteronuclear cross relaxation.^53^

**Figure 2:**
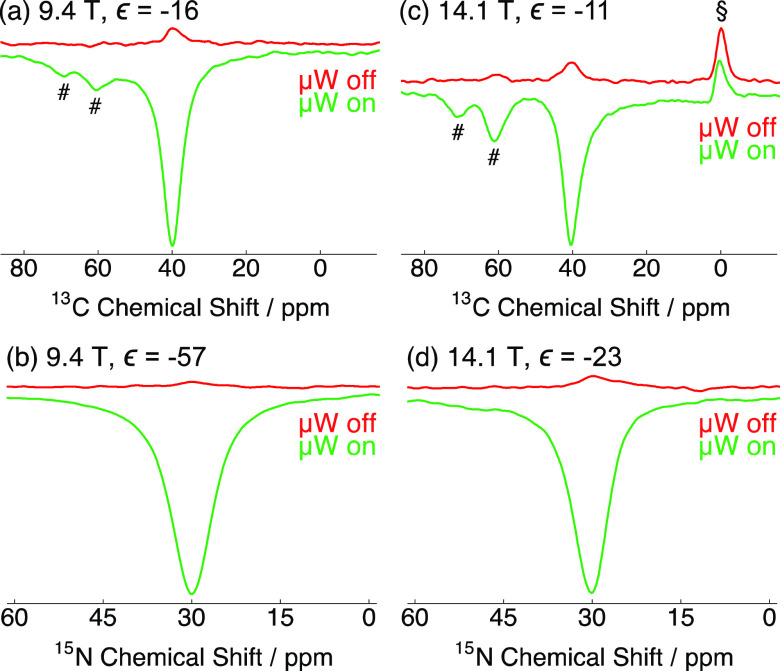
Relevant portions of the experimental (a, c) ^13^C and (b, d) ^15^N NMR spectra of 1.5 M [2-^13^C,^15^N]glycine doped with 20 mM Gd(NO_3_)_3_·6H_2_O dissolved in H_2_O/D_2_O/glycerol-*d*_8_ (1/3/6 v/v/v) acquired at (a, b) 9.4 T and (c, d) 14.1 T and ∼105 K without (red) and with (green) microwaves (μW) at the optimum negative positions of the NMR signal enhancement profiles (see Figure 1). Spectra were obtained by direct excitation. # and § indicate ^13^C NMR peaks belonging to glycereol-*d*_8_ and the ^13^C NMR peak of the silicone plug used in experiments at 14.1 T, respectively.

^13^C nuclear spins show experimental NMR signal enhancements of ϵ = −16 and −11 at 9.4 and 14.1 T, respectively, and were recorded at the negative lobe of the ^13^C Zeeman field profiles in Figure 1 (hence the opposite phase vs the microwave off NMR spectra). This is more clearly demonstrated by considering the ^13^C NMR spectra shown in Figure 2a and c, where, in both cases, the ^13^C NMR signal from the ^13^C-labeled 2-position site of glycine (∼40 ppm) is strongly negatively enhanced. The ^13^C NMR peaks belonging to natural abundance glycerol-*d*_8_ (∼60–70 ppm) are also enhanced, while that of the silicone plug (at 14.1 T) remains thermally polarized and of positive phase.

Significant experimental NMR signal enhancements are observed for ^15^N nuclear spins, with a boost in ^15^N NMR signal intensity by a factor of ϵ = −57 at 9.4 T and ϵ = −23 at 14.1 T also recorded at the negative lobe of the ^15^N Zeeman field profiles. The ^15^N NMR signal from the ^15^N-labeled site of glycine (∼30 ppm), which is barely visible in the microwave off spectrum at 9.4 T, is now clearly observable in the ^15^N NMR spectra presented in Figure 2b and d. This is obtained with satisfactory signal-to-noise ratios (SNRs) in a reasonable short time frame of ca. 5 min.

Table 1 presents all values of ϵ and ϵ over the square root of the polarization buildup time constants under microwave irradiation *T*_B,ON_ at 9.4 and 14.1 T. Table 1 also provides a measure of the overall NMR signal enhancement ϵ_θ_,^48^ a more representative parameter to report the actual sensitivity gain with respect to a non-DNP formulation.^16^ Quantification of ϵ_θ_ is given by
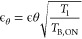
1where θ is the contribution from paramagnetic quenching^48^ (θ = 0.77 in our experiments) and *T*_1_ is the nuclear spin–lattice relaxation time constant of an undoped frozen solution. We note here that, to the best of our knowledge, the ^13^C and ^15^N *T*_1_ values of glycine are reported here for the first time in a frozen solution at ∼105 K (see Table S2 and the Methods section for more details). In eq 1, experimental time savings are considered, which ultimately leads to higher values of DNP signal enhancements, particularly for lower gamma nuclear spins where the value of *T*_1_ can be excessively long. In our experiments at 9.4 T (Table 1), ϵ_θ_ = −35 and ϵ_θ_ = −197 for ^13^C and ^15^N were achieved, respectively.

**Figure 3:**
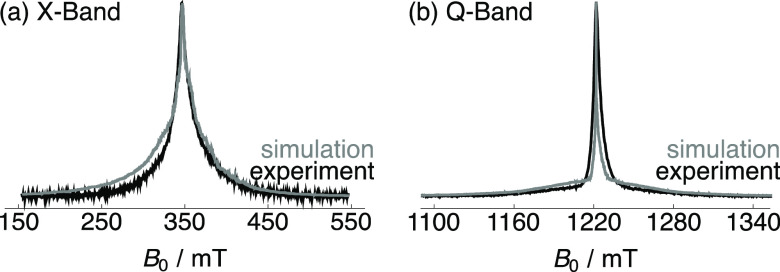
Comparison of the relevant portions of the experimental echo-detected EPR spectra (black) of 20 mM Gd(NO_3_)_3_·6H_2_O dissolved in H_2_O/glycerol (2/3 v/v) acquired at (a) X-band (9.5 GHz) and (b) Q-band (34 GHz) and 100 K with simulated echo-detected EPR spectra (gray) for *D* = 810 ± 90 MHz, σ_D_ = *D*/3, and *g* = 1.98510 (see the main text for more details). The fwhm of the central transition is ∼20 mT at X-band and ∼6 mT at Q-band.

Figure 3 presents the experimental echo-detected EPR spectra (black) of 20 mM Gd(NO_3_)_3_·6H_2_O dissolved in H_2_O/glycerol (2/3 v/v) acquired at X-band (9.5 GHz, Figure 3a) and Q-band (34 GHz, Figure 3b) and 100 K. The corresponding simulated echo-detected EPR spectra (gray in Figure 3) were obtained with the EPR software package EasySpin^52^ and were reasonably reproduced using a value of *D* = 810 ± 90 MHz for the axial component of the ZFS interaction with a Gaussian distribution σ_D_ = *D*/3^54^ (Figure S4) and a *g*-factor of *g* = 1.98510. Electron spin relaxation time constants were measured on the same sample under the same conditions and yield *T*_1e_ = 319 ± 1 ns and *T*_2e_ = 72 ± 1 ns at X-band.

## Discussion

Gd^3+^ is a high-spin paramagnetic metal ion with a total spin *S* = 7/2 and a 4f^7^ electronic configuration carrying zero orbital angular momentum with *g* ≃ 2 as determined from EPR spectral simulation. We therefore assume that the EPR line width is determined by the central (*m*_S_ = −1/2 ↔ +1/2) transition of the Gd^3+^ electron spin, i.e., a pseudo spin-1/2 system, and that the corresponding full-width at half-maximum (fwhm) Δ_h_ is broadened by second-order effects scaling as
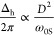
2where ω_0S_ is the electron Larmor frequency, while neglecting all other EPR line shape broadening contributions such as electron-dipolar couplings and hyperfine interactions.^54^ In addition, the ZFS interaction directly influences all single quantum satellite transitions (*m*_S_ = ± 1/2 ↔ ±3/2, ±3/2 ↔ ±5/2, ±5/2 ↔ ±7/2) and is a perturbation to the Zeeman interaction, which leads to a broad EPR spectrum with a sharp, central resonance (as seen in Figure 3b). Furthermore, hyperfine interactions with ^155^Gd and ^157^Gd nuclear spins (ca. 15% natural isotopic abundance) also contribute to EPR line broadening.

The DNP profiles shown in Figure 1 indicate that the SE mechanism is predominantly responsible for polarization transfer. The SE mechanism can be modeled by considering a two-spin-1/2 system including one electron spin and one nuclear spin. It is known that the two-spin process yields evolution of the involved nuclear spin polarization *P*_I_ upon activation of the microwave source:^55^
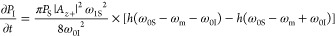
3where *P*_S_ is the electron spin polarization level, *A*_*z*+_ is the superhyperfine interaction constant between the electron and nuclear spins, ω_1S_ and ω_m_ are the amplitude and frequency of the microwave field, respectively, and ω_0I_ is the nuclear Larmor frequency. The overall line shape function *h*(ω_0S_ – ω_m_ ± ω_0I_) describing the EPR spectrum includes both homogeneous and inhomogeneous contributions to the EPR spectral line width, which is modeled by Lorentzian profiles centered at ω_0S_ ± ω_0I_:
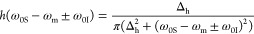
4where Δ_h_ has been defined above.

The negative lobes of the ^1^H NMR Zeeman field signal profile acquired at either 9.4 or 14.1 T were well fitted using a Lorentzian function of the kind given by eq 4 with an additional linear slope to account for non-negligible baseline distortions (Figure S2). Fits of the negative lobes at both 9.4 and 14.1 T returned values of Δ_h_ = 25 ± 2 MHz and 27 ± 6 MHz, respectively. The values for Δ_h_ indicate very narrow NMR signal enhancement profiles,^40^ which are reflected by the EPR line shapes (Figure 3) and are also within error of those previously reported in the literature for other Gd^3+^-containing PAs.^41,43^

The solid lines in Figure 1 (and Figure S1 for a full ^1^H detected Zeeman profile) are representative of eq 3, which return NMR Zeeman field profiles with positive maxima and negative minima separated by 2ω_0I_ as experimentally observed for ^1^H (Figure S1) and ^15^N (Figure 1b); that is, the NMR signal enhancement profiles are consistent with the SE DNP mechanism being mostly responsible for polarization transfer. In all cases, the factor of  was adjusted to vertically scale the experimental and theoretical curves. Due to the limited maximum current permitted in the sweep coil of the NMR magnet that defines the upper limits of *B*_0_ (indicated by the vertical dashed lines in Figure 1), only the negative lobes of the Zeeman field profiles were accessed for ^1^H (14.1 T), ^13^C (both fields), and ^15^N (9.4 T). Given that in the SE DNP mechanism the positive lobe is approximately symmetrical vs ω_0S_, there is no obvious requirement for the use of higher *B*_0_ fields. Such commercial DNP spectrometers are designed with a specific build to enable sweep capabilities with sufficient magnetic field stability (even after a sweep) for most samples at low temperature where inhomogeneous broadening can be severe.^49^ There is no necessary requirement to choose a fixed *B*_0_, which would otherwise compromise obtainable NMR signal enhancements for all investigated nuclear spins.

The *S* = 7/2 quantum number introduces several other energy levels, anisotropic broadening mechanisms, and relaxation (particularly prevalent at high PA concentrations) which might be present in the DNP process and complicate further analysis. Moreover, the description presented above does not consider the influence of MAS, the rhombicity of the ZFS interaction, or a distribution of ZFS parameters.^54^ Nevertheless, the simplifications discussed above yield remarkably good agreement between the experimental DNP enhancement profiles and the simulated curves. It should also be noted that although homogeneous broadening is likely to be small compared with the broadening induced by the ZFS, dipolar broadening due to the 20 mM concentration of the Gd ion source may introduce a significant Lorentzian component to the DNP field profiles and, as a result, a Lorentzian function appears to sufficiently fit the DNP field profiles. However, a minor deviation from pure SE is observed for the case of ^13^C nuclear spins between experimental and theoretical curves toward the center of the NMR Zeeman field profiles. This manifests as a broad component present in the central portion of the ^13^C NMR signal enhancement profile as observed for [Gd(dota)(H_2_O)]^−^.^43^ This is attributed to the cross effect DNP mechanism,^41^ which is also partly observable for ^15^N nuclear spins (Figure 1b).

The experimental echo-detected EPR spectra shown in Figure 3 are reasonably well simulated using a value of *D* = 810 ± 90 MHz. This value is larger than those recently reported in the literature for other gadolinium complexes (e.g., 410 MHz for [Gd(tpcatcn)] and 599 MHz for [Gd(dota)(H_2_O)]^−^ complexes),^43^ but is closer to that reported for GdCl_3_ (784 MHz).^41^ This is expected since the hydration shells are strongly related for the two Gd salt PAs (see below). Gd(NO_3_)_3_ exists in pure aqueous solution (below pH ≃ 6) as a hydrated Gd(H_2_O)_8–9_^3+^ ion with ∼8–9 inner-sphere water molecules^56^ and a square antiprism/tricapped trigonal prism geometry with the gadolinium ion at the center. High-resolution mass spectrometry (HRMS) data obtained on Gd(NO_3_)_3_·6H_2_O and GdCl_3_·6H_2_O dissolved in H_2_O/glycerol (2/3 v/v) suggest [Gd(glycerol)_2_(NO_3_)]^2+^ and [Gd(glycerol)_2_]^3+^ fragments, respectively, probably with highly labile inner-sphere H_2_O ligands and thus suggest a different ligand sphere. Cl^–^ is a polarizable anion and has weak affinity for the Gd^3+^ ion. In aqueous media, Cl^–^ does not likely enter the inner coordination sphere of the Gd^3+^ ion and only forms a weak outer-sphere complex. On the other hand, NO_3_^–^ is a donor (and bidentate) that is more likely to bind to the inner coordination sphere of the Gd^3+^ ion, even in the presence of water. To the best of our knowledge, there is only one example of the coordination chemistry of glycerol with a single series of lanthanide complexes,^57^ and glycerol is shown to act as both bidentate and tridentate ligands. This is illustrated in Figure S5 for postulated Gd^3+^ lanthanide ion coordination geometries with a highly unsymmetrical metal ion environment.

The central transition of the experimental EPR spectrum is slightly broader than the simulated EPR spectrum at X-band, indicating a potentially larger value of the ZFS for Gd(NO_3_)_3_·6H_2_O. However, it is worth noting that the use of a fully protonated DNP solvent increases the number of electron–proton dipole–dipole interactions compared with using DNP juice, and a 20 mM concentration of Gd(NO_3_)_3_·6H_2_O results in electron–electron dipole–dipole interactions of increased strength, both of which contribute to broadening of the EPR line and would lead to a partial overestimation of *D*, with the latter being the more significant EPR line broadening mechanism in this case. The spectra also indicate that the EPR line width is mostly dominated by the ZFS interaction, as opposed to homogeneous line broadening contributions, since there is a significant reduction in EPR line width (ca. 14 mT) when moving from X-band to Q-band.

Recently, several gadolinium-based PAs have been developed for MAS DNP NMR experiments with a specific set of design principles in mind.^44^ It was concluded that signal enhancements from gadolinium PAs are inversely proportional to *D*^2^, and so a lower value of *D* leads to larger NMR signal enhancements, as powerfully demonstrated from the synthetic design of the structural complex [Gd(tpcatcn)].^43^ The value of *D* measured and the enhancement factors ϵ obtained in this work compare favorably with those of Gd(tpcatcn), [Gd(dota)(H_2_O)]^−^, and GdCl_3_, from the previous literature^41,43^ and the empirical reciprocal quadratic relationship.^44^ The value of *D* for the ZFS obtained from the EPR spectrum of GdCl_3_·6H_2_O is marginally smaller (by a factor of ∼1.14) than the *D* reported in this work for the ZFS of Gd(NO_3_)_3_·6H_2_O. A broad background signal was present in the EPR spectrum of GdCl_3_, which was attributed to a second complex with a much larger ZFS.^41^ Furthermore, GdCl_3_ was tested as a PA for DNP at 5 T, the efficiency of which was found to be approximately half of that of [Gd(dota)(H_2_O)]^−^. In the same work, [Gd(dota)(H_2_O)]^−^ was also trialled at 9.4 T for direct ^13^C and ^15^N DNP. The enhancement factors reported in this work are ∼2.5 and ∼2.2 times smaller, respectively. These findings indicate that the DNP efficiencies of Gd(NO_3_)_3_ and GdCl_3_ are of a similar order of magnitude. This is likely related to the relative values of *D* reported for the ZFS of the two off-the-shelf Gd ion sources.

A comparison of DNP efficiencies between different Gd PAs is also related to the line widths of the central transitions obtained from the DNP profiles. The line width of the central transition strongly depends on the distribution of values of the ZFS, which is known to be nontrivial for Gd complexes.^56^ Central transition line widths of 23 and 22 MHz were observed^41^ for [Gd(dota)(H_2_O)]^−^ at 9.4 and 14.1 T, respectively, which are narrower than the central transition line widths reported in this work (Figure 3). This result also supports the conclusion that Gd(NO_3_)_3_ has a larger ZFS constant than both [Gd(dota)(H_2_O)]^−^ and [Gd(tpcatcn)].

Electron spin relaxation is usually attenuated at 100 K compared with room temperature, and the elongated *T*_1e_ and *T*_2e_ relaxation time constants are typically suitable for favorable MAS DNP enhancements.^58,59^ Electron spin relaxation of Gd complexes is dependent on concentration, magnetic field, and temperature. The electron spin relaxation time constants of 25 mM [Gd(dota)(H_2_O)]^−^ were calculated to be *T*_1e_ = 1.66 μs and *T*_2e_ = 1.81 ns at X-band and ∼113 K.^60^ The value of *T*_1e_ reported in this work for Gd(NO_3_)_3_ is a factor of ∼5 shorter, while the value of *T*_2e_ is considerably longer (a factor of ∼40). The shortened value of *T*_1e_ has only a minor influence on the DNP efficiency of Gd(NO_3_)_3_ vs other Gd-complexed PAs, e.g., [Gd(dota)(H_2_O)]^−^. However, the longer value of *T*_2e_ is expected to give rise to improved enhancements vs [Gd(dota)(H_2_O)]^−^, which are not observed in this work. This may be associated with the 20 mM Gd(NO_3_)_3_·6H_2_O concentration used in our experiments and/or the absence of ligands stabilizing the Gd center.

The DNP enhancement factors decrease with an increasing magnetic field, showing that there is clearly a trade-off between DNP signal enhancement due to the SE polarization transfer mechanism becoming more significant at lower magnetic fields and the narrowing of the central transition at higher magnetic fields.^54^ The observed ^13^C and ^15^N NMR signal enhancements are expected to primarily result from direct DNP rather than relayed transfers by spin diffusion, as this contribution is not anticipated to be large for the ^13^C and ^15^N nuclear spins. The polarization buildup times *T*_B,ON_ are reported in Table 1, and their stretched exponential nature (see the Methods section for more details) relates to a distribution for the values of *D*.^61^ Values for ϵ, ϵ_θ_, and for  (see Table 1) are significantly greater for ^15^N and ^13^C than ^1^H, highlighting the benefit of the approach for lower gamma nuclear spins.

## Conclusions

We have reported Gd(NO_3_)_3_·6H_2_O as an efficient PA for MAS DNP NMR experiments with an experimental NMR signal enhancement of −57 being achieved for the ^15^N nuclear spins of [2-^13^C,^15^N]glycine at 9.4 T and ∼105 K. The NMR signal enhancement profiles obtained at 9.4 and 14.1 T are indicative of SE DNP, which is supported by the EPR data. These encouraging results are exciting for future development of paramagnetic metal ions as PAs in MAS DNP experiments, particularly for direct polarization of lower gamma nuclear spins, such as ^13^C and ^15^N, and complement data previously acquired on Gd complexes with tailor-designed ligands. This work was not intended to identify best-in-class Gd ion PAs, but rather to illustrate enhanced NMR sensitivity without the need for any synthetic chemistry given that Gd(NO_3_)_3_·6H_2_O is readily available, easily affordable, and chemically neutral and stable under a range of conditions. Improving the observed NMR signal enhancements could be achieved by further sample formulation optimization (including reducing paramagnetic bleaching). In particular, solutions of Gd(NO_3_)_3_ could be employed to formulate solid materials (including microcrystalline solids) by incipient wetness impregnation.^8,15^ The value of *g* in Gd(NO_3_)_3_ is suitable as a PA for SE DNP at moderate magnetic fields and suggests that other paramagnetic (transition) metal ions in related compounds could act as suitable PAs and are currently being explored.

## Supporting Information Available

Experimental ^1^H Zeeman field profile at 9.4 T, magnified views of the negative lobes of the ^1^H via CP Zeeman field profiles, additional experimental ^13^C CP DNP MAS NMR spectra, simulated EPR spectra as a function of *D*, proposed chemical structure of Gd(NO_3_)_3_·6H_2_O dissolved in H_2_O/D_2_O/glycerol-*d*_8_ (1/3/6 v/v/v), and fitted relaxation data (PDF)
