# Supplementary material for: Off-the-Shelf Gd(NO3)3 as an Efficient High-Spin Metal Ion Polarizing Agent for Magic Angle Spinning Dynamic Nuclear Polarization
Source: J Phys Chem B. 2022 Aug 16;126(33):6281–9. doi: 10.1021/acs.jpcb.2c04184 (PMC9421651; doi:10.1021/acs.jpcb.2c04184)
Supplement: Supplementary file 1 — jp2c04184_si_001.pdf [file jp2c04184_si_001.pdf]

Supporting Information for

Off-the-shelf $Gd(NO_3)_3$ as an Efficient High-Spin Metal Ion Polarising Agent for Magic Angle Spinning Dynamic Nuclear Polarisation

Stuart J. Elliott[a,b], Benjamin B. Duff[a,c], Ashlea R. Taylor-Hughes[a], Daniel J. Cheney[a], John P. Corley[a], Subhradip Paul[d,e], Adam Brookfield[f], Shane Pawsey[g], David Gajan[h], Helen C. Aspinall[a], Anne Lesage[c] and Frédéric Blanc[a,c,*]

[a] Department of Chemistry, University of Liverpool, Liverpool L69 7ZD, United Kingdom
[b] Current Address: Molecular Sciences Research Hub, Imperial College London, London W12 0BZ, United Kingdom
[c] Stephenson Institute for Renewable Energy, University of Liverpool, Liverpool L69 7ZD, United Kingdom
[d] DNP MAS NMR Facility, Sir Peter Mansfield Imaging Centre, University of Nottingham, Nottingham NG7 3RD, United Kingdom
[e] Current Address: CEA, IRIG, MEM, 38000 Grenoble, France
[f] Department of Chemistry and Photon Science Institute, University of Manchester, Oxford Road, Manchester M13 9PL, United Kingdom
[g] Bruker BioSpin Corporation, Billerica, MA 01821, United States of America
[h] Université de Lyon, Centre de Résonance Magnétique Nucléaire à Très Hauts Champs (UMR 5082, CNRS/ENS Lyon/UCBL), 69100 Villeurbanne, France

* Correspondence should be addressed to frederic.blanc@liverpool.ac.uk

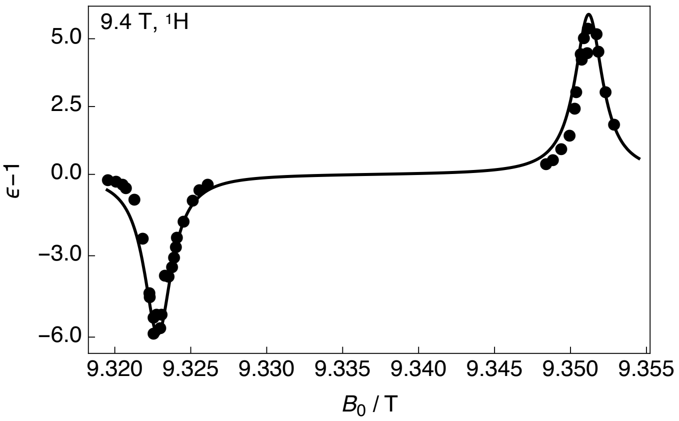

Figure S1: Normalized experimental ^{1}H DNP MAS NMR Zeeman field profiles of 1.5 M [2-^{13}C,^{15}N]glycine doped with 20 mM $Gd(NO_3)_3 \cdot 6H_2O$ dissolved in H_2O/D_2O/glycerol-*d*$_8$ (1/3/6 *v*/*v*/*v*) as a function of the static magnetic field (B_0) acquired at 9.4 T and ~105 K. The vertical axes are given as normalized enhancements (ϵ-1). Solid lines are theoretical curves (see Equation 2 and the main text for more details). The frequency separation between the positive maximum and negative minimum is ~$2\omega_{0S}$. Note that this dataset was recorded on a 400 MHz system with a slightly lower B_0 field which shifts the upper limits of B_0 and enables the positive maximum to also be observed (*vs.* Figure 1 of the main text). The ^{1}H enhancements are given by direct ^{1}H detection and not via ^{13}C CP. In this case, the choice of a 10 s DNP build-up time is responsible for the increased signal enhancements *vs.* the ones shown in the main text.

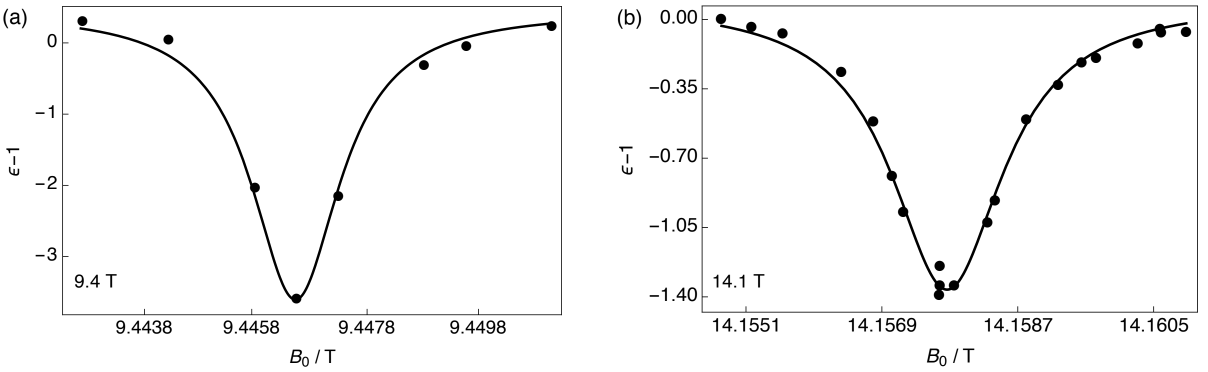

Figure S2: Experimental (black dots) and simulation (solid lines) of the negative lobes of the ^{1}H via CP Zeeman field profiles of 1.5 M [2-^{13}C,^{15}N]glycine doped with 20 mM $Gd(NO_3)_3 \cdot 6H_2O$ dissolved in H_2O/D_2O/glycerol-*d*$_8$ (1/3/6 *v*/*v*/*v*) at (a) 9.4 T and (b) 14.1 T. The simulation is based on the Lorentzian function given by Equation 3 of the main text and returned best fit values for the corresponding full-width half-maximum (FWHM) Δ_h of 27 ± 6 MHz at 9.4 T and 25 ± 2 MHz at 14.1 T.

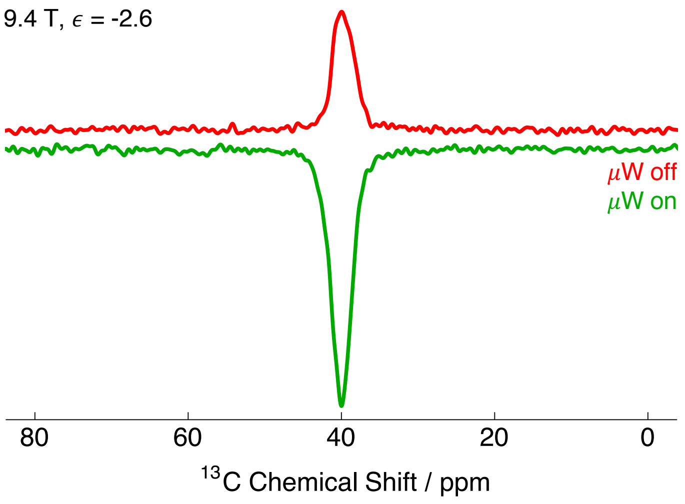

Figure S3: Experimental ^{13}C CP DNP MAS NMR spectra of 1.5 M [2-^{13}C,^{15}N]glycine doped with 20 mM $Gd(NO_3)_3{\cdot}6H_2O$ dissolved in H_2O/D_2O/glycerol-d_8 (1/3/6 *v*/*v*/*v*) acquired at 9.4 T and ~105 K without (red) and with (green) microwaves (μW) at the optimum negative position of the ^1H NMR signal enhancement profile (see Figure 1).

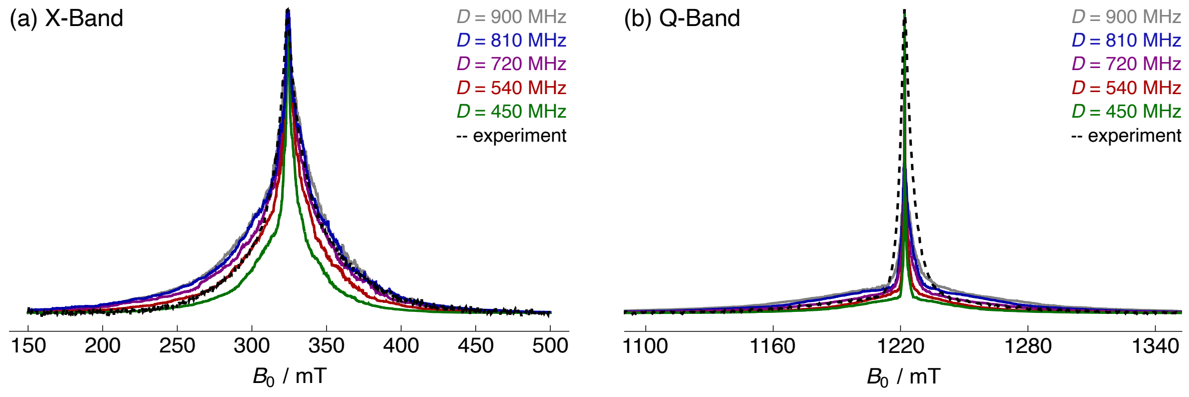

Figure S4: Comparison of the relevant portions of the experimental echo-detected EPR spectra (black) of 20 mM $Gd(NO_3)_3{\cdot}6H_2O$ dissolved in H_2O/glycerol (2/3 *v*/*v*) acquired at (a) X-band (9.5 GHz) and (b) Q-band (34 GHz) and 100 K with simulated echo-detected EPR spectra (green, red, purple, blue and grey) for various values of *D* with σ_D = *D*/3 and g = 1.98510 (see the main text for more details).

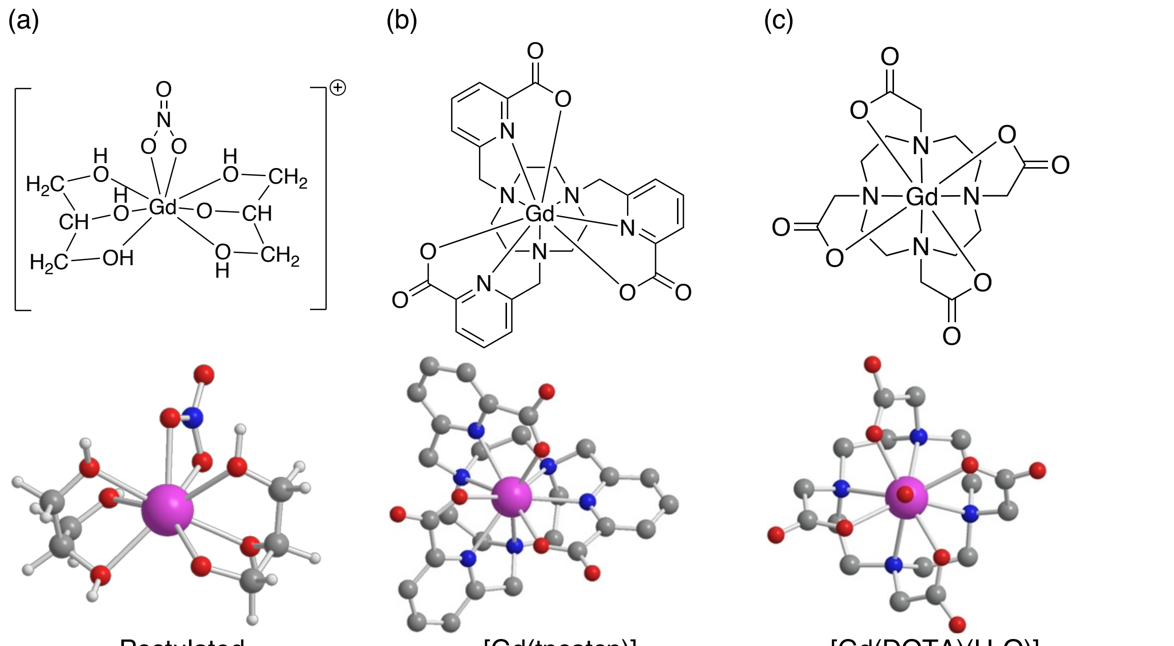

Postulated [Gd(H_3glyc)(H_2glyc)(NO_3)]$^+$ complex (H_3glyc = glycerol)

[Gd(tpcatcn)]

[Gd(DOTA)(H_2O)]$^-$

Figure S5: Proposed chemical structures of (a) $Gd(NO_3)_3 \cdot 6H_2O$ dissolved in H_2O/glycerol (2/3 *v*/*v*) based on the literature[1] and which gives an idea of how the molecules of glycerol fit around the Gd^{3+} centre, compared with structures of (b) [Gd(tpcatcn)][2] and (c) [Gd(dota)(H_2O)]$^-$[3].

Table S1: Complete set of fitted relaxation parameters for the DNP build-up time constants $T_{\mathrm{B,ON}}$ for ^{1}H, ^{13}C and ^{15}N nuclear spins at 9.4 and 14.1 T. 1.5 M [2-^{13}C,^{15}N]glycine doped with 20 mM $Gd(NO_3)_3{\cdot}6H_2O$ in the glass-forming mixture H_2O/D_2O/glycerol-d_8 (1/3/6 *v*/*v*/*v* ratio) at ~105 K was used. Data were fitted to A(1-exp{-$(t/T^{*}_{\mathrm{B,ON}})^{\alpha}$}), where A is a fitting constant, $T_{\mathrm{B,ON}} = T^{*}_{\mathrm{B,ON}}\Gamma(1/\alpha)/\alpha$, $T^{*}_{\mathrm{B,ON}}$ is the DNP build-up time constant extracted from the above-described fitting procedure, α is the breadth of the distribution of DNP build-up time constants and $\Gamma(1/\alpha)$ is the gamma function.

	$T_{\mathrm{B,ON}}$ / s			A			α		
B_0 / T	^{1}H	^{13}C	^{15}N	^{1}H	^{13}C	^{15}N	^{1}H	^{13}C	^{15}N
9.4	3.6 ± 0.1	165 ± 25	240 ± 11	0.98 ± 0.01	0.93 ± 0.04	0.98 ± 0.01	1.08 ± 0.03	0.82 ± 0.08	0.65 ± 0.01
14.1	14 ± 2	222 ± 7	304 ± 31	0.95 ± 0.03	0.99 ± 0.01	0.98 ± 0.02	1.75 ± 0.05	0.91 ± 0.02	0.63 ± 0.01

Table S2: Complete set of fitted relaxation parameters for the nuclear spin-lattice relaxation time constants T_1 for ^{1}H, ^{13}C and ^{15}N nuclear spins at 9.4 T. 1.5 M [2-^{13}C,^{15}N]glycine in the glass-forming mixture H_2O/D_2O/glycerol-d_8 (1/3/6 *v*/*v*/*v* ratio) at ~105 K was used. ^{1}H relaxation data were fit with an exponential function of the type B(1-exp{-t/T_1}) where B is a fitting constant. ^{13}C/^{15}N relaxation data were fit with a bi-exponential function of the type Cexp{-t/T_1}+Dexp{-t/$T_{i,r}$} where C and D are fitting constants and $T_{i,r}$ accounts for an initial, rapid decay of nuclear magnetisation.

T_1 / s			$T_{\mathrm{i,r}}$ / s		B	C		D	
^{1}H	^{13}C	^{15}N	^{13}C	^{15}N	^{1}H	^{13}C	^{15}N	^{13}C	^{15}N
66.1 ± 0.5	1338 ± 87	4882 ± 419	19.0 ± 2.4	121 ± 133	1.11 ± 0.01	0.88 ± 0.01	0.87 ± 0.05	0.12 ± 0.01	0.15 ± 0.06

Table S3: Complete set of fitted relaxation parameters for the electron spin longitudinal and transverse relaxation times T_{1e} and T_{2e} at X-band. 20 mM $Gd(NO_3)_3{\cdot}6H_2O$ in the glass-forming mixture H_2O/glycerol (2/3 *v*/*v* ratio) at ~105 K was used. T_{2e} and T_{1e} were fitted to E(1-exp{-(t/T_{2e})}) and F(1-exp{-$(t/T^{*}_{1e})^{\beta}$}), where E and F are fitting constants, $T_{1e} = T^{*}_{1e}\Gamma(1/\beta)/\beta$, T^{*}_{1e} is the electron longitudinal relaxation time constants extracted from the above-described fitting procedure, β is the breadth of longitudinal relaxation time constants and $\Gamma(1/\beta)$ is the s function.

T_{2e} / ns	E	T_{1e} / ns	F	β
72 ± 1	1.05± 0.01	319 ± 1	1.00 ± 0.01	0.74 ± 0.01

References

(1) Naumov, N. G.; Tarasenko, M. S.; Virovets, A. V.; Kim, Y.; Kim, S.; Fedorov, V. E. Glycerol as Ligand: The Synthesis, Crystal Structure, and Properties of Compounds $[Ln_2(H_2L)_2(H_3L)_4][Re_6Q_8(CN)_6]$, Ln = La, Nd, Gd, Q = S, Se. *Eur. J. Inorg. Chem.* **2006**, *2006*, 298–303.

(2) Gateau, C.; Mazzanti, M.; Pécaut, J.; Dunand, F. A.; Helm, L. Solid-State and Solution Properties of the Lanthanide Complexes of a New Nonadentate Tripodal Ligand Derived from 1,4,7-Triazacyclononane. *Dalt. Trans.* **2003**, 2428–2433.

(3) Chang, C. A.; Francesconi, L. C.; Malley, M. F.; Kumar, K.; Gougoutas, J. Z.; Tweedle, M. F.; Lee, D. W.; Wilson, L. J. Synthesis, Characterization, and Crystal Structures of M(DO3A) (M = Iron, Gadolinium) and Na[M(DOTA)] (M = Fe, Yttrium, Gd). *Inorg. Chem.* **1993**, *32*, 3501–3508.
